# The Changing Factors Affecting Local Environmental Governance in China: Evidence from a Study of Prefecture-Level Cities in Guangdong Province

**DOI:** 10.3390/ijerph17103573

**Published:** 2020-05-20

**Authors:** Jiangmin Yang, Desheng Xue, Gengzhi Huang

**Affiliations:** 1 Guangdong Provincial Key Laboratory for Urbanization and Geo-Simulation, School of Geography and Planning, Sun Yat-sen University, Guangzhou 501275, China; yangjm7@mail2.sysu.edu.cn; 2 Southern Marine Science and Engineering Guangdong Laboratory (Zhuhai), Zhuhai 519082, China

**Keywords:** environmental governance, local government, global–local interactions, geographical detector, China

## Abstract

This paper aims to examine the changing factors underlying China’s environmental governance, by investigating the long-term dynamic impacts of related endogenous and exogenous factors and considering regional differences in these factors. The study estimated environmental regulation intensity and employed the geographical detector technique to analyze its driving factors, based on 21 prefecture-level cities in Guangdong Province, China, from 1990 to 2016. The results showed that environmental regulation intensity has increased in Guangdong Province over the past 27 years. The results also indicated that local environmental governance was affected by global–local interactions and changed based on different regional developmental phases. At first, factors within a region affected local environmental policies more significantly, such as economic development and urbanization. However, in the long run, globalization’s impacts have been the most important factors influencing environmental governance. Further analysis showed that environmental regulation intensity in Guangdong Province’s different regions was affected by different driven factors. Our analyses contribute to the understanding of China’s environmental governance and have policy implications for environmental problem management and China’s construction of an ecological civilization.

## 1. Introduction

After China’s reform and opening up, the country faced an unprecedented economic boost that made it the largest developing country in the world [1]. Simultaneously, such extensive development came at the cost of remarkable resource consumption, which exacerbated the deterioration of the ecological environment [2,3]. In 2018, the air pollution index of 64.2% of cities exceeded the standard, while 55.3% of land did not achieve good environmental quality, based the China Ecological Environment Bulletin [4]. Facing severe environmental problems, environmental elements have gradually become a key consideration in Chinese urban governance; meanwhile, environmental governance has played an increasing role in urban and regional development [5]. To improve environmental quality, in recent years, a series of environmental policies and measures based on local features has been undertaken by the Chinese government. 

Following the trend of neoliberalization in most developed countries, the government’s role has weakened, while non-state actors have had more opportunities to influence environmental governance [6,7]. In contrast with the Western model of neoliberal governance, which emphasizes individualism over collective responsibility, the leading role in formulating and implementing environmental regulations in China has always been taken on by different governmental levels; non-state actors in China mainly depend on government authority to exert their influence in managing environmental problems [8,9]. Additionally, local governments play a vital role in solving local environmental problems by responding to the policies of national and provincial governments [10,11,12].

This paper contributes to the literature by integrating the different impacts from endogenous and exogenous factors on local environmental management into a comprehensive understanding of environmental governance in China. Although many case and country studies have demonstrated how such factors contribute to environmental governance, few studies have examined the influence of global-local interactions on the management of environmental problems in the Chinese context specifically. Additionally, in practice, there is little empirical evidence regarding how changing factors affect environmental governance in the Chinese context in the long run, or how these impacts vary among regions.

To fill this gap in the literature, this study investigates the long-term dynamic impacts of endogenous and exogenous factors on China’s environmental governance, by analyzing panel data of 21 prefecture-level cities in Guangdong Province from 1990 to 2016. Environmental regulation intensity was calculated to examine local environmental governance’s changes at the prefecture level in Guangdong from 1990 to 2016. Additionally, the geographical detector technique was used to estimate the driving forces of urban environmental governance. Specifically, this study sought to answer the following two related questions: (1) what drives the changes of local environmental governance in different periods in Guangdong Province, China? (2) How do these impacts change in developed and developing regions? The study found that the impact of endogenous and exogenous factors on local environmental governance follows a global–local pattern, and the impact varies across different regions. Moreover, the paper concludes by suggesting that both the different developmental phases cities undergo and the global–local context should be considered in future environmental governance and sustainable development policies in China.

The remainder of this study is structured as follows. Following a brief literature review in Section 2, Section 3 explains the materials and methods used. Section 4 presents the statistical results and discusses the possible reasons behind them. Section 5 concludes with a summary of the main findings. Finally, this paper provides some policy suggestions.

## 2. Literature Review

### 2.1. Regional Development and Environmental Governance

Economic globalization has a profound impact on the development of cities and regions, and has involved great changes with regard to the path regional development has taken and its main actors [13,14]. In addition to the influence of endogenous factors within a region, scholars argue that regional development should be considered from the perspective of global–local interactions, to combine the basic interregional impacts with those of globalization [15,16]. 

Detrimental environmental impacts in regional contexts from human activities have gained attention. Some studies have focused on the relationship between environmental quality and economic growth in different regional contexts, and on different indicators measuring environmental quality [17,18,19,20]. These studies have found that regional economic development led to environmental pollution under certain conditions. Additionally, the acceleration of industrialization and globalization has aggravated the deterioration of the ecological environment [21,22]. 

Although economic growth still remains the priority for different levels of government, there is an increasing imperative to move toward more sustainable models of regional development through a growing number of policy initiatives. The sixth Global Environment Outlook, published by the United Nations Environment Programme in 2019, indicated the importance of environmental governance, and called for urgent and more aggressive action to protect the environment [23]. Some researchers have claimed that environmental governance should be used as a tool to enhance regional performance and promote sustainable development [24,25]. The implementation of environmental governance in reducing regional air pollution and promoting regional sustainable development have been proved effective in some studies [26]. Thus, it is argued that environmental governance plays a vital role in dealing with the environmental issues caused by the regional development under the impact of global–local interactions. 

### 2.2. Factors of Environmental Governance

Notably, regional environmental conditions are one of the critical factors affecting human survival, as well as regional development. Increasingly, studies have contributed to an understanding of the key factors of local environmental governance behavior that aims to achieve a good ecological environment. They have provided contextualized evidence as to why cities and regions have dealt with environmental problems, but have yielded inconsistent findings and complicated the understanding of environmental governance. 

On one hand, scholars have been investigating the influence of regional endogenous factors on environmental governance. For example, Peng et al. [27] argued that a series of social and economic factors, including economic level, urban construction, industrial structure, and population density, have impacted urban environmental governance in China based on their analysis of eight cities on the Yangtze River. However, Cale and Reams [28] found that in the U.S., social and economic factors did not have a significant influence on environmental regulation. Other studies have found that civic education played a role in the participation of citizens in environmental policy, promoting environmental governance and sustainable development [29,30]. Moreover, other research has considered the environmental impact of cooperation with neighboring cities. For instance, some researchers have found that environmental regulations in neighboring areas could encourage enhanced environmental governance of other counties and cities [28].

On the other hand, scholars have also been concerned with exogenous factors’ effects and have claimed that globalization has contributed to strengthening local environmental protection capacity and promoting sustainability; that is, it is not purely an economical phenomenon [31]. For instance, empirical studies have shown a positive correlation between economic and social globalization and the quality of governance [32]. Kurniawan et al. [33] further indicated that international cooperation between the Global North and South has improved the environmental governance of cities in developing countries through the transfer of capital and technology. In China, Yang et al. [34] found that globalization has had an important impact on environmental governance, improving urban environmental quality in Guangdong Province.

To summarize, the nature of certain factors’ influence on environmental governance is inconclusive and debatable. Thus, this paper contributes to the literature in two ways. First, it provides empirical evidence from China to enrich the understanding of its environmental governance and the factors that influence it. Although earnest efforts have been made to investigate the driving factors of local environmental governance behavior, little scholarly attention has been given to China—the biggest developing country, and one whose enforcement model follows a top-down direction, which differs from neoliberal environmental governance. Given the inconclusive nature of research on environmental governance, changes in environmental governance and the corresponding driving factors in China have yet to be verified. Second, this paper enriches scholars’ understanding of environmental governance using a geographical perspective by examining how its driving factors differ in diverse regions of Guangdong Province, one of the most developed provinces in China. Although China, as a socialist country, has begun to tackle the problem of uneven development, regional developmental and environmental disparities are prominent. Unlike existing research, which is limited to national-level data, this paper examined Guangdong Province by disaggregating it into two regions, developed and developing, to introduce some nuance and geographical understanding into the research regarding factors influencing local environmental governance in the Chinese context. 

## 3. Materials and Methods

### 3.1. Case Study Area: Guangdong Province, China

**Figure 1:**
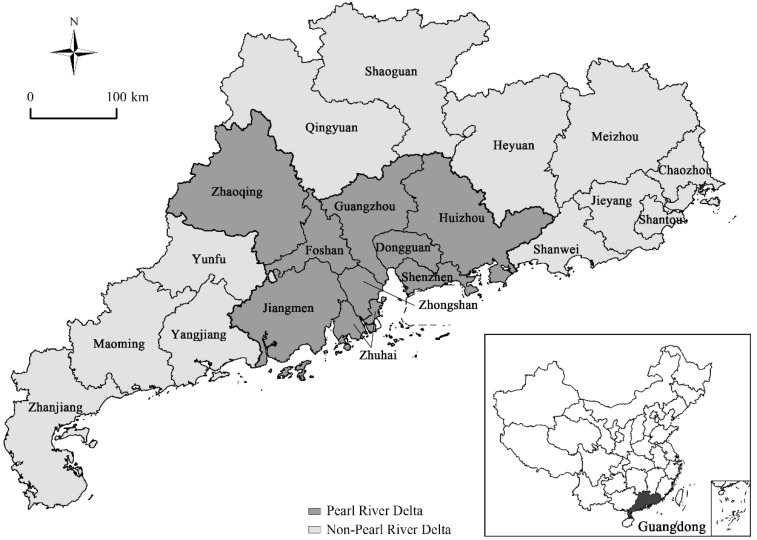
Location and regional divisions of Guangdong Province. The figure is based on the image downloaded from the website of the National Administration of Surveying, Mapping and Geoinformation (http://bzdt.ch.mnr.gov.cn/).

Located in southeastern China, Guangdong Province has experienced unprecedented economic growth and has become the most developed province in China, with the gross domestic product of 8555.57 billion yuan in 2016, because of the reform and opening-up policies. Additionally, it is China’s most populous province, with a resident population that reached 109.99 million in 2016, up from 61.04 million in 1990, and has a land area of 179.7 thousand square km. Economic development and population growth in this region followed a core-periphery gradient, with the core Pearl River Delta (PRD) as the developed region and the peripheral Non-Pearl River Delta (NPRD) as the developing region, as shown in Figure 1 [35]. Moreover, this regional difference in Guangdong Province mirrors that of the eastern, central, and western regions of China. Rapid development of pollution-intensive industries in Guangdong, which has become “the factory of the world,” badly damaged the environment. Foreign investment in China has greatly increased since the l990s, aggravating the environmental degradation. However, the current environmental quality is greatly improved, due to the implementation of strict environmental measures, such as green development, making Guangdong the benchmark for other provinces regarding environmental efficiency [36]. Guangdong’s development path is the epitome of the “growth first, cleaning up later” philosophy of China [37]. Undoubtedly, Guangdong Province is highly representative of China as a whole, in terms of regional differences and environmental management, especially in the context of globalization, and its environmental governance process since 1990 deserves more attention.

### 3.2. Materials

#### 3.2.1. Data Sources

This study used the data of 21 prefecture-level cities in the PRD and NPRD regions of Guangdong Province from 1990 to 2016. The data on these cities were derived mainly from the China City Statistical Yearbook, Guangdong Statistical Yearbook, China Exhibition Annuals, and China’s Ports-of-Entry Yearbook. Every effort was made to obtain data for a single index in different years from the same source to ensure accuracy and comparability. Additionally, for missing data, data from the Statistical Yearbook of the prefecture-level cities were used as a replacement value. It should be clarified that the pollution emissions of cities from 1990 to 1995 were taken from a compilation of environmental statistics released by the Department of Ecology and Environment of Guangdong Province. The rest of the pollution emission data from 1996 to 2016 were derived from the Guangdong Statistical Yearbook, and the statistical yearbooks did not include data on pollution emissions in the cities of Guangdong Province after 2016. Thus, it was not possible in this study to examine the intensity of environmental regulation in each city after 2016. Although the sources of the pollution emission data are different between the two periods, 1990–1995 and 1996–2016, this had little influence on the process of examining the changing factors affecting local environmental governance. The figures and tables in Section 4 were based on the empirical results found in this study. 

#### 3.2.2. Factor Acquisitions

Previous studies have discussed the effects of factors within and outside a given region and concluded that these have important but different impacts on cities’ environmental governance. Thus, based on the concept framework of global-local interactions [15,16], this study chose 10 endogenous and exogenous factors and investigated their impacts on the environmental governance of cities (Table 1). The five endogenous factors are four representations of cities’ social and economic features—economic growth (GDP), industrial structure (TI), urbanization (UI), and citizen quality (EDU)—and one indicator of cooperation between neighbor cities within regions—environmental cooperation (NER). The five exogenous factors are the amount of international twin cities (CITY), first-class open ports (OPEN), realized foreign investments (FDI), projects for contracted foreign direct investment (ITEM), and international tourists (TOUR); these were used to measure the levels of governmental globalization, economic globalization, and globalization of social communications.

### 3.3. Methods

#### 3.3.1. Environmental Regulation Intensity

Existing research has used various methods to calculate environmental governance, which can be divided into two categories. Some research is based on the perspective of regulation cost, such as investment in environmental pollution control and cost of pollution treatment facilities [38,39]. Additionally, many studies have focused on regulation effects using the measurement of pollutant emission density to represent environmental regulation intensity [40,41]. Based on the principles of appropriateness, comparability, and accessibility, this paper uses the second measure, pollutant emission density, to calculate the extent of environmental governance mainly enforced by local governments in China. The basic idea of this method is that the paper first constructed the relative positions of different kinds of pollutant emission intensity throughout the whole province, and then took a weighted average of the relative levels of all kinds of pollution emission intensity in individual cities to investigate the efforts of environmental pollution control made by those cities using the following formula [40]:

(1) Pollution emissions intensity of city *i*
  (1) $$P_{\mathrm{ijt}}=\frac{\frac{T_{\mathrm{ijt}}}{Y_{\mathrm{it}}}}{\frac{1}{21}\sum_{i=1}^{21}\frac{T_{\mathrm{ijt}}}{Y_{\mathrm{it}}}}$$
  where *i*, *t*, and *j* stand for city, year, and pollutant, respectively; *P_ijt_* is the pollution emissions intensity of pollutant *j* in city *i* during year *t*. *T_ijt_* is the total discharge of pollutant *j* in city *i* during year *t*. *Y_it_* is the gross industrial output value in city *i* during year *t*. The higher value of *P_ijt_*, especially if it exceeds one, indicates that the pollution emissions of cities are relatively higher in the province, due to conduction of looser environmental regulation.
(2) Total pollution emissions intensity of city *i*
  (2) $$P_{\mathrm{it}}=\frac{1}{3}{(P}_{i1t}{+P}_{i2t}{+P}_{i3t})$$
  where *P_it_* is the total pollution emissions intensity in city *i* during year *t* as measured based on industrial wastewater, industrial sulfur dioxide, and industrial soot (dust). Industrial pollution is the main source of pollution in China and includes wastewater, gaseous waste, and solid waste. This study considered the three main types of industrial pollution, industrial wastewater, industrial sulfur dioxide, and industrial soot (dust), which are more frequently used in the existing research and easily accessed in the Statistical Yearbook. Since *P_ijt_* is dimensionless, it makes sense to calculate the total pollution emissions intensity mathematically, as the units do not need to be standardized.
(3) Environmental regulation intensity of city *i*
  (3) $${\mathrm{ER}}_{\mathrm{it}}=\frac{1}{P_{\mathrm{it}}}$$
  where *ER_it_* is the environmental regulation intensity in city *i* during year *t*. The higher value of *ER_it_* indicates that cities achieve better environmental governance by conducting stricter environmental regulation; otherwise, the environmental standards and environmental regulation intensity are lower.

#### 3.3.2. Gini Coefficient

The Gini coefficient is employed first to mirror the wealth gap in a country or region, and then to reflect the difference between cities within a region [42]. In this study, the Gini coefficient was used to analyze the regional difference of environmental regulation intensity in Guangdong Province with the following formula [42]:

(4) $$G=\frac{2\times \sum_{i=1}^{n}{i\;\times \;x}_{i}}{n^{2}{\;\times \;u}_{x}}-\frac{n+1}{n}$$

where *G* is the Gini coefficient, *n* is the number of cities, *x_i_* is the environmental regulation intensity of city *i*, and *u_x_* is the average environmental regulation intensity of all cities. Within the range of values 0–1, the higher the Gini coefficient, the more unbalanced its distribution.

#### 3.3.3. Geographical Detector

Geographical detector is used to test the coupling between two variables based on their spatial distributions [43]. In this study, the influence mechanisms underlying changes of environmental regulation intensity were analyzed using the factor detector and interaction detector based on Geodetector software (http://www.geodetector.cn/). The factor detector can be used to measure the determinant power of influencing factors on environmental regulation intensity; its model is as follows [43]:

(5) $$q_{D,H}=1-\frac{1}{{n\sigma }_{H}^{2}}\sum_{i=1}^{m}n_{D,i}\sigma_{H_{D,j}}^{2}$$

where *q_D,H_* is the detection force index of influencing factors on the change of environmental regulation intensity, *D* represents the influencing factor, *n* is the number of samples in the whole area, *m* is the number of strata for a given factor, $\sigma_{H}^{2}$ represents the variance of environmental regulation intensity of the whole area, and $\sigma_{H_{D,j}}^{2}$ represents the variance of secondary regions. Note that $\sigma_{H_{D,j}}^{2}$ ≠ 0 and the range of *q_D,H_* is 0 to 1. The higher value of *q_D,H_* suggests the greater influence of the factor *D* on the change of environmental regulation intensity.

**Figure A1:**
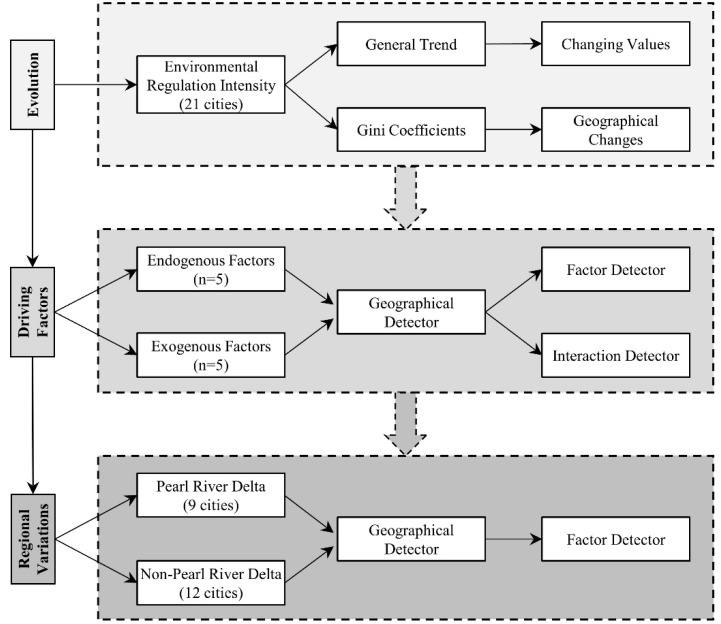
Flowchart of the Methodological Strategy.

The interaction detector was used to analyze the effect of the interaction of two or more factors on the change of environmental regulation intensity. Two influencing factors may be independent or have a combined effect (Table 2) [43]. If there is a combined effect, the effect of these factors will be greater after intersecting. The steps used in all of the methods in this study are presented in a flowchart (Figure A1).

## 4. Results and Discussion

### 4.1. Characteristics of the Evolution of Environmental Regulation Intensity

**Figure 2:**
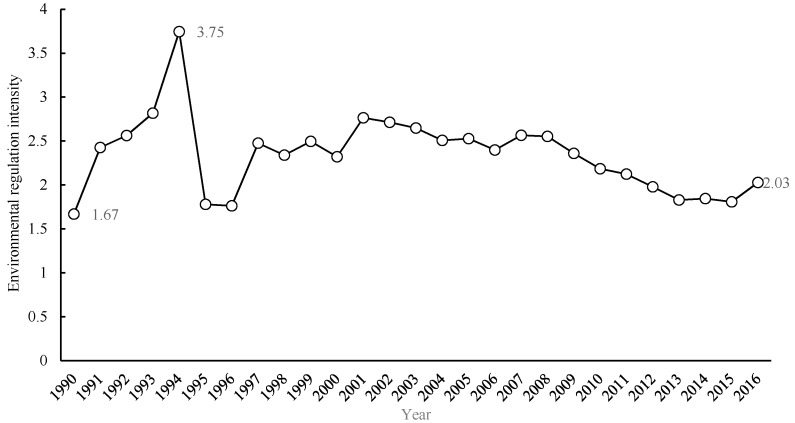
Changing trends of average environmental regulation in Guangdong Province.

To identify the general trend of environmental regulation intensity in Guangdong Province, this study computed the environmental regulation intensity for 21 cities from 1990 to 2016. It used the full sample of cities to calculate the annual means for Guangdong Province, as shown in Figure 2. The statistics show that environmental regulation intensity in Guangdong has consistently increased from 1.67 to 2.03 over the past 27 years, and can be divided into two phases. During the first phase, from 1990 to 2000, environmental regulation intensity changed frequently, meaning that pollution emissions heavily contaminated the environment, and city governments enforced additional stricter environmental policies for several years. The environmental regulation intensity increased rapidly from 1990 to 1994, and reached the peak of 3.75 in 1994. This was because a series of environmental policies were implemented to strengthen environmental governance. Notably, China’s Agenda 21 was finished in 1993, and was put into effect to pursue sustainable development in 1994. The progress of the control project as a part of the annual environmental protection plan, which was listed in the China Economic and Society Development Plan for the first time in 1994, was remarkable. This national policy event may have had a direct but short-lived impact on local environmental regulation, leading to stronger but temporary environmental regulation in Guangdong in the 90s. However, the facts showed that the impact of the policy was subject to the goal of economic growth, which was overriding. Even so, during the second phase after 2000, environmental regulation intensity was reduced slightly but remained stable, indicating that the end-of-pipe control of environmental pollution was effective and environmental pollution levels were relaxing in Guangdong. In 2012, the construction of ecological civilization was proposed, aimed toward the coordination of the ecological environment and economic development in China, similar to the cooperation between municipalities and national parks in Poland, one of the European countries of the former Eastern Bloc [44]. Additionally, more attention was paid to pollution prevention and ecological protection, which played a positive role in promoting sustainable development. The positive effects of environmental governance in China contrast with the impact of government intervention on agricultural development in Poland [45]. 

**Figure 3:**
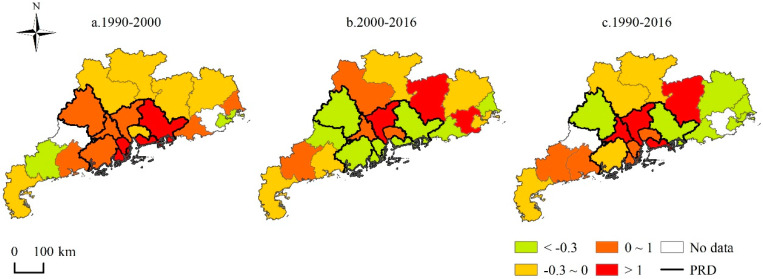
Changing values of the environmental regulation intensity of cities, 1990–2016.

Changing environmental regulation intensity in the different periods, 1990–2000, 2000–2016, and 1990–2016, can be broken down and further compared (Figure 3). From 1990 to 2016, the values of environmental regulation intensity of seven cities (corresponding to 33.3% of all cities) decreased by 0.3, which indicates that most cities’ environmental regulations have increased in intensity in Guangdong Province over the past 27 years. Additionally, the environmental regulation intensity of four cities showed an increase of over 1, focused in the PRD region. Between 1990 and 2000, the environmental regulation intensity of most cities increased; only eight cities loosened their environmental management. However, only seven cities increased their environmental regulation intensity from 2000 to 2016. As a whole, the increase of cities’ environmental regulation intensity over the past 27 years mainly occurred during the first phase, before 2000. 

**Figure 4:**
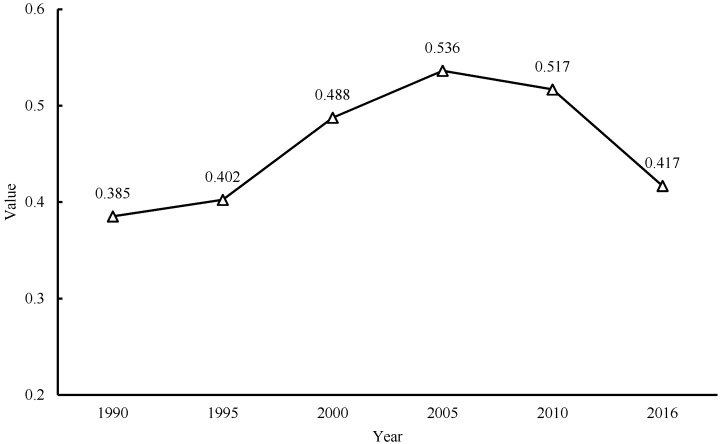
Gini coefficients of environmental regulation intensity in Guangdong Province, 1990–2016.

Geographical analysis showed that cities’ environmental regulation intensity was distributed unevenly across Guangdong Province. The Gini coefficients of environmental regulation intensity in Guangdong Province were calculated in the selected years from 1990 to 2016 (Figure 4). The results show that the Gini coefficient increased during the period of 1990 to 2016, reaching the peak of 0.536 in 2005, and then decreased slightly to 0.417 by 2016. This indicates that the regional difference of environmental regulation intensity in Guangdong Province is significant and extremely sizable, although it has been narrowing since 2005.

**Figure 5:**
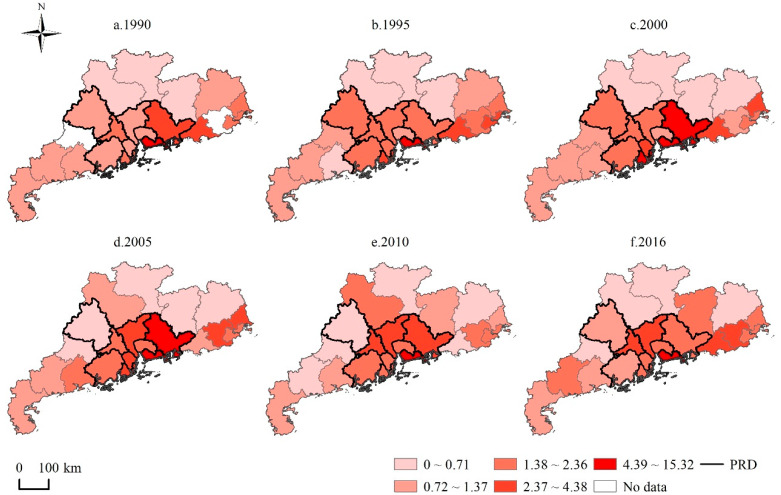
Spatial discrepancies of environmental regulation intensity in Guangdong Province, 1990–2016.

Each city’s environmental regulation intensity from 1990 to 2016 was classified into five levels, using the natural breaks method, as shown in Figure 5. The environmental regulation intensity of cities along the coast was higher than those in the inland area. Additionally, the environmental regulation intensity for most PRD cities was much higher than in the NPRD region, especially after 2000, which indicates that environmental governance in the PRD region was stricter than in the NPRD region. Additionally, as Figure 5 shows, the environmental regulation intensity of Guangzhou, Foshan, Heyuan, and Dongguan increased significantly from 1990 to 2016, especially for Guangzhou and Foshan, which increased from level 2 to level 4. In addition, Shenzhen’s environmental governance was ranked the highest among the cities in Guangdong Province, meaning it enforced the most rigorous environmental policies. 

### 4.2. Driving Factors of Environmental Regulation Intensity

#### 4.2.1. Dominant Factors of Environmental Regulation Intensity

To identify the driving forces of changes in environmental regulation intensity and check whether two influencing factors work independently or not, this study assessed the factor detector and interaction detector of the 21 cities for 1990–2016. Before Geodetector analysis, a discrete transformation was used to transform all data using IBM SPSS Statistics version 18.0. The respective results for factor and interaction detectors are presented in Table 3 and Table 4. 

Table 3 shows that the five endogenous factors (GDP, TI, EDU, UI and NER), and the five exogenous factors (CITY, OPEN, FDI, ITEM, and TOUR), influenced environmental regulation intensity (ER) significantly between 1990 and 2016. Additionally, among the factors, the two maximal detection and explanatory power *q* results for ER from 1990 to 2016 were 0.25 (ITEM) and 0.21 (OPEN). The two most important factors influencing environmental regulation based on the explanatory power *q* among the five endogenous factors were GDP (0.13) and UI (0.13). 

The empirical results indicate that urban sustainability is closely related to the social and economic characteristics of cities in China, and these characteristics differ from those found by research in the U.S. [28]. Among these factors, economic growth and urbanization have important effects on environmental governance of cities in Guangdong Province. Rapid economic growth provided significant funding and technological support for enforcing environmental governance. The significant relationship between urbanization and environmental regulation is represented in two ways. First, urbanization improved the efficiency of resource utilization and the effectiveness of pollution control [46]. Second, urbanization brought more attention to public health [47], leading to the need for local governments to improve the living environment by implementing effective environmental policies. 

Furthermore, economic and governmental globalization played a leading role in influencing local environmental governance in Guangdong Province. In the early stage after the reform and opening-up in 1978, governments in Guangdong lowered environmental standards to attract pollution-intensive industries from developed countries [41]. However, with the deepening of economic globalization, governments made efforts to improve the environment by enforcing stricter environmental governance, aiming to attract high-quality foreign direct investment and accelerate economic growth. During this process, environmental governance benefited from the advanced technologies and upgraded industries introduced to Guangdong Province through globalization [34,48]. Moreover, broad global communication and cooperation between cities in terms of politics, economics, and environmental protection promoted environmental governance in Guangdong Province in formal and informal ways.

Table 4 indicates that only two types of interaction between any two factors occurred, namely nonlinear enhancement and bi-enhancement, indicating that the explanatory power of any two influencing factors’ interaction was greater than that of a single factor. The interactions of ITEM with GDP (0.4279) and that of ITEM with OPEN (0.4238) had the strongest explanatory power for ER, suggesting the interactions among globalization, economic growth, and other factors were the most impactful for environmental governance in the cities of Guangdong Province. These results further demonstrate that local environmental governance was affected by interactions among multiple endogenous and exogenous factors.

Overall, the endogenous and exogenous factors and their interactions exerted significant effects on the environmental governance of 21 cities in Guangdong Province.

#### 4.2.2. Changing Factors of Environmental Regulation Intensity

The respective results using the factor detector are presented in Table 5 and are sorted by the values of explanatory power *q* to compare their changing effects in different periods: 1990–1999, 2000–2009, and 2010–2016. 

Table 5 shows that GDP (0.21) and UI (0.18) were the top two factors influencing ER from 1990 to 1999, suggesting that economic growth and urbanization were the leading causes of the changing environmental regulation intensity in the early stage before 2000. However, the top three factors that impacted ER in the next two periods, 2000–2009 and 2010–2106, were ITEM (0.43; 0.51), CITY (0.41; 0.38), and FDI (0.37; 0.32), which indicates that the impact of exogenous factors gradually exceeded that of endogenous ones, and they became the main driving factors of local environmental governance in Guangdong Province.

The explanatory power *q* of exogenous factors increased overall, and their rank increased significantly over time. For example, the impact CITY was not significant in the first period (1990–1999), but its explanatory power *q* increased to 0.38, and it became the second maximal detector for ER in the third period (2010–2016). Moreover, the rank of ITEM was upgraded from seventh in the first period to first in the last period. This indicates that economic and government globalization had an increasing effect on cities’ environmental governance.

By contrast, the explanatory power *q* of endogenous factors also increased gradually but decreased in rank. Even though GDP’s explanatory power *q* grew from 0.21 to 0.28 from the first to the last period, it went from being the maximal detector for ER in the first period. to the sixth highest factor in the last period, which suggests that with changes in developmental goals, unchecked economic development’s constraining force on environmental governance weakened. Moreover, EDU’s explanatory power *q* increased from 0.06 to 0.17, corresponding to the lowest effect on environmental governance in Guangdong Province since 2000.

Our results are consistent with the situation of Guangdong Province. On one hand, this is because in the early part of the study’s time period, economic development was the cities’ main goal, and local governments prioritized urbanization [49,50], leading to its increased impact on environmental governance. In the meantime, better environmental quality through enforcement of environmental governance may improve economic development and urbanization further. With the changes in developmental goals, local governments began to focus more on the construction of an ecological civilization, the coordination of the ecological environment and economic development, rather than on the overriding goal of economic growth, which led to the evolution of the conception of environmental management [51,52]. Thus, the impact of economic growth and urbanization on local environmental governance decreased.

On the other hand, notably, since China became a member of the World Trade Organization, globalization’s influence has accelerated greatly, increasing its impact on urban development, including economic growth, industrial structure, technological advancement, and relevant environmental standards [33,46]. Motivated by the omnidirectional, deep impacts of globalization, higher environmental standards from more access to international communication led city governments to change environmental policies and promote sustainable development [53,54]. 

### 4.3. Regional Variations of Driving Factors Influencing Environmental Regulation Intensity

Regions and cities have various degrees of development and are affected differently by endogenous and exogenous factors, so they may show diverse influencing effects. The estimated results of the factor detection method (Table 6) used in this study confirm that local environmental governance in different regions in Guangdong Province was affected by different factors.

Table 6 shows that GDP, EDU, UI, NER, FDI, ITEM, and TOUR all had significant effects on ER in both the PRD and NPRD regions, which indicates that the endogenous and exogenous factors impacted environmental regulation at the regional level. This is consistent with the trend seen at the province level. However, notable differences exist in the relationship between environmental regulation intensity and these factors, which are caused by different interactions of multiple factors in the two regions.

As seen in Table 6, CITY and OPEN did not have a statistically significant impact on ER in the NPRD region; this is because there are few international twin cities and first-class open ports in this region. Additionally, the exogenous factors had a larger impact, and OPEN and ITEM played a leading role in enhancing environmental governance in the highly developed PRD region. This further confirms that the exogenous effects on environmental governance exceeded the endogenous ones when considering cities’ long-term globalization process and comparing the different developmental phases of the two regions. Additionally, NER had the greatest influence in the NPRD region, indicating environmental cooperation is needed in a developing region to improve the environment. Moreover, TI had a nonsignificant impact on environmental regulation in the NPRD region. In the long run, more optimized industrial structure and advanced technology use in the economy can be expected, causing strengthened governance for environmental issues. Thus, it can be concluded that the environmental governance in the developed PRD region is mainly impacted by the globalization-driven pattern but is under the localization-driven mode in the less developed NPRD region.

## 5. Conclusions

This study’s goal was to examine the changing factors of local environmental governance in Guangdong Province, China, and to consider regional differences among these factors by using calculations of the environmental regulation intensity and data of 21 prefecture-level cities from 1990 to 2016 with the geographical detector technique. This paper contributes to furthering the understanding of China’s environmental governance by indicating the different factors of governments’ environmental management during this period within the context of global–local interactions.

The empirical results reveal that for 27 years, environmental regulation intensity has increased in Guangdong Province. Environmental regulation intensity increased greatly from 1990 to 2000, while after 2000, it was stable. Additionally, there is a significant difference between the PRD and NPRD regions regarding the implementation of environmental regulation, owing to the different developmental phases the regions experienced. 

This study also finds that environmental governance that is mainly enforced by local governments in Guangdong Province results from the interactions of endogenous and exogenous factors. Moreover, the main driving factors of environmental governance depend on the characteristics of different developmental phases. At first, endogenous factors, such as economic growth and urbanization exerted the main influence from 1990 to 2000. However, exogenous factors have become the primary influence over time, especially in terms of the impact from economic and governmental globalization. After 2000, cities were strongly affected by global factors, and local governments aimed to follow the trend of globalization, contributing to the impacts on environmental governance. Thus, cities’ different developmental phases and global–local context should be one of the most important considerations when seeking to understand the characteristics and driving factors of local environmental governance in China.

This paper has enriched the debate surrounding environmental governance and its influencing factors from a longer-term, geographical perspective, by introducing regional nuance into the existing evidence regarding China. It has revealed that endogenous and exogenous factors have important effects on environmental regulation intensity across both regions of Guangdong Province included in this study, and that regional differences in this relationship are caused by different interactions of multiple factors. It also suggests the following three interesting conclusions. First, globalization has a stronger impact on environmental governance in the developed PRD region, compared with the less developed NPRD region. Second, environmental cooperation plays a leading role in enhancing environmental regulation in the NPRD region. Third, the effect of international twin cities and first-class open ports on the management of environmental problems in the NPRD region is not statistically significant—probably because the majority of this region is inland, so international cooperation and exchange through international cities and major ports is limited. 

Finally, some methodological limitations need to be addressed to better understand the changes of environmental governance and their related factors. First, how to more precisely estimate environmental governance remains a challenge for current research. This paper calculated environmental regulation intensity by searching previous studies and collecting available municipal-level data. Thus, different indexes and a finer geographical level are needed for future studies. Second, owing to the nature of statistical analysis, the complex interactions between local environmental governance and its driving factors could not be explained in detail; thus, exemplary cases could be used to support our findings in future research. Third, since the latest statistical yearbooks did not release the data on pollution emissions in the cities of Guangdong Province, this study was unable to examine the intensity of environmental regulation in each city after 2016; this is necessary in future research. Given the limitations of this study, future studies should consider addressing these issues and providing a more in-depth discussion. 

## 6. Policy Implications

This paper has several policy implications. First, its findings suggest that global-local impacts should be included in policy frameworks for sustainable development and the Chinese government’s ecological civilization project. Second, because of current inadequate intercity environmental cooperation, local governments in developed regions should offer financial and technological assistance to cities in developing regions, thus contributing to the enhancement of their environmental standards and environmental pollution treatment technologies. Last, based on basic requirements for environmental protection in China, spatial differences between cities and regions should be taken into consideration when making specific environmental protection policies. Given the different development phases that affect environmental governance in different regions, an elastic, region-based environmental management system should be enforced.

## Figures and Tables

**Table 1:** Definitions of variables.

Category	Variable	Definitions	Abbreviation
Endogenous factors	Economic growth	Per capita GDP (RMB yuan)	*GDP*
	Industrial structure	Percentage of secondary industry in GDP (%)	*TI*
	Urbanization	Percentage of urban population in permanent residents (%)	*UI*
	Citizen quality	Student enrollment in regular institutions of higher education (person)	*EDU*
	Environmental cooperation	Intensity mean of environmental regulation of neighbor cities	*NER*
Exogenous factors	Governmental globalization	International twin cities	*CITY*
		First-class open ports	*OPEN*
	Economic globalization	Realized foreign investments (10,000 dollars)	*FDI*
		Projects for contracted foreign direct investment	*ITEM*
	Social communication globalization	International tourists (10,000 persons)	*TOUR*

**Table 2:** Redefined interaction relationships.

Description	Interaction
*q*(*X*_1_ ∩ *X*_2_) < Min(*q*(*X*_1_), *q*(*X*_2_))	Weaken, nonlinear
Min(*q*(*X*_1_), *q*(*X*_2_)) < *q*(*X*_1_ ∩ *X*_2_) < Max(*q*(*X*_1_), *q*(*X*_2_))	Weaken, uni-
*q*(*X*_1_ ∩ *X*_2_) > Max(*q*(*X*_1_), *q*(*X*_2_))	Enhance, bi-
*q*(*X*_1_ ∩ *X*_2_) = *q*(*X*_1_) + *q*(*X*_2_)	Independent
*q*(*X*_1_ ∩ *X*_2_) > *q*(*X*_1_) + *q*(*X*_2_)	Enhance, nonlinear

**Table 3:** Factor detection results of environmental regulation intensity in Guangdong Province, 1990–2016.

Factors	*GDP*	*TI*	*EDU*	*UI*	*NER*	*CITY*	*OPEN*	*FDI*	*ITEM*	*TOUR*
*q* statistic	0.13 ***	0.11 ***	0.05 ***	0.13 ***	0.11 ***	0.17 ***	0.21 ***	0.20 ***	0.25 ***	0.17 ***
*p* value	0.00	0.00	0.00	0.00	0.00	0.00	0.00	0.00	0.00	0.00

Note: *, ** and *** indicate rejection of the null hypothesis at the 10%, 5% and 1% significance levels, respectively.

**Table 4:** Interaction detection results of environmental regulation intensity in Guangdong Province, 1990–2016.

Factors	GDP	TI	EDU	BUD	NER	CITY	OPEN	FDI	ITEM	TOUR
*GDP*	0.1270									
*TI*	0.2202	0.1116								
*EDU*	0.2941	0.2010	0.0456							
*BUD*	0.2860	0.2553	0.3931	0.1267						
*NER*	0.3193	0.2914	0.3008	0.3485	0.1071					
*CITY*	0.2346	0.2147	0.3149	0.3079	0.2956	0.1717				
*OPEN*	0.2799	0.2719	0.3298	0.3624	0.3474	0.2772	0.2068			
*FDI*	0.2953	0.2918	0.3018	0.3285	0.3855	0.2762	0.2859	0.2017		
*ITEM*	0.4279	0.3453	0.3679	0.4050	0.4171	0.3663	0.4238	0.3436	0.2502	
*TOUR*	0.2319	0.2191	0.2944	0.2929	0.3452	0.2175	0.2614	0.2673	0.4079	0.1744

**Table 5:** Factor detection results of environmental regulation intensity in three periods in Guangdong Province.

1990–1999		2000–2009		2010–2016	
Factors	*q*	Factors	*q*	Factors	*q*
*GDP*	0.21 ***	*ITEM*	0.43 ***	*ITEM*	0.51 ***
*UI*	0.18 ***	*CITY*	0.42 ***	*CITY*	0.38 ***
*OPEN*	0.16 ***	*FDI*	0.37 ***	*FDI*	0.32 ***
*TOUR*	0.15 ***	*TOUR*	0.29 ***	*OPEN*	0.29 ***
*NER*	0.11 ***	*GDP*	0.28 ***	*UI*	0.29 ***
*FDI*	0.10 **	*TI*	0.26 ***	*GDP*	0.28 ***
*ITEM*	0.09 **	*OPEN*	0.25 ***	*TI*	0.21 ***
*TI*	0.08 *	*UI*	0.21 ***	*NER*	0.20 ***
*CITY*	0.07	*NER*	0.20 ***	*TOUR*	0.19 ***
*EDU*	0.06	*EDU*	0.10 ***	*EDU*	0.17 ***

Note: *, ** and *** indicate rejection of the null hypothesis at the 10%, 5% and 1% significance levels, respectively.

**Table 6:** Factor detection results of environmental regulation intensity in different regions of Guangdong Province, 1990–2016.

Factors	*GDP*	*TI*	*EDU*	*UI*	*NER*	*CITY*	*OPEN*	*FDI*	*ITEM*	*TOUR*
PRD	0.10 ***	0.09 **	0.07 ***	0.13 ***	0.19 ***	0.14 **	0.28 ***	0.23 ***	0.25 ***	0.23 ***
NPRD	0.03 *	0.02	0.05 ***	0.07 ***	0.10 ***	0.02	0.01	0.04 **	0.07 ***	0.04 *

Note: *, ** and *** indicate rejection of the null hypothesis at the 10%, 5% and 1% significance levels, respectively.
